# Availability of Cognitive Remediation Therapy in United States Mental Health Facilities Serving Older Adults: A Statistical Study

**DOI:** 10.7759/cureus.81187

**Published:** 2025-03-25

**Authors:** Tajudeen O Basiru, Oluwatosin O Arubuolawe, Oluwaseun Sonola, Chidalu Ibeneme, Charlene S Abiodun, Charles c Nnamchi, Kemsyriochukwu Ogala, Rheiner N Mbaezue, Salisu Aikoye, Beatriz D'Onghia

**Affiliations:** 1 Behavioral Health, Community Health of South Florida, Miami, USA; 2 Psychiatry and Behavioral Sciences, Manhattan Psychiatric Center, New York, USA; 3 Crisis Intervention, Cornwall Community Hospital, Cornwall, CAN; 4 Public Health, University of Toledo, Toledo, USA; 5 Psychiatry, All Saints University School of Medicine, Roseau, DMA; 6 Behavioral Health, Houston Behavioral Healthcare Hospital, Houston, USA; 7 Psychiatry, Sagemont Preparatory School, Weston, USA; 8 Mental Health, University of Wisconsin, Wisconsin, USA; 9 Psychiatry, Charles Drew University, Los Angeles, USA

**Keywords:** crt, mental health, older adults, psr, samhsa, vcr

## Abstract

Introduction: Older age is a major risk factor for many neurocognitive disorders like Alzheimer's disease. While there are limited treatment options for improving memory in this population, research has shown that special psychotherapeutic modalities like Cognitive Remediation Therapy (CRT), especially when coupled with psychosocial and vocational rehabilitation (PSR and VCR respectively), benefit older adults. This study examines the availability of these services in US mental health (MH) facilities serving older adults and compares the characteristics of the facilities providing these services.

Methods: Our study includes 1216 MH facilities using the 2022 National Substance Use and Mental Health Services Survey (N-SUMHSS) data from the Substance Abuse and Mental Health Services Administration (SAMHSA). Descriptive statistics were used to determine availabilities, while multivariable logistic regression was used to compare facilities that offer the services compared to those that do not.

Results: Of the total facilities included, 78 (6%), 634 (52%), and 381 (31%) offered CRT, PSR, and VCR respectively. Facilities that provided only MH services compared to those providing substance use and MH services, those that offer special services for veterans, and special Alzheimer’s programs compared to those that do not were more likely to have CRT services. MH facilities that provide supplemental employment services, housing services and recovery housing were more likely to offer PSR and VCR compared to those that do not. Compared to facilities in Midwest states, facilities in East South-Central were less likely to offer PSR and VCR.

Conclusion: This study highlights the relative unavailability of CRT, indicating a need for more interest in evidence-based nonpharmacological treatment options for cognitive decline in the aging population.

## Introduction

Older age is a significant risk factor for many neurocognitive disorders like Alzheimer's disease. The proportion of the US population 65 years and older has been growing lately, according to the World Population Prospects 2022 [1], increasing interest in their physical and mental health. Cognitive decline is associated with older age [2] and is a major component of many neurocognitive disorders that are prevalent in old age, such as Alzheimer's disease and vascular dementia [3]. Although cardiovascular risk factors like hypertension, diabetes mellitus, hyperlipidemia, and smoking, as well as psychosocial factors like social isolation, psychological stress, anxiety, or depression, are strongly linked to neurocognitive disorders [4], age is considered the most critical risk factor associated with cognitive dysfunction as well as neurocognitive disorders. The burden posed by cognitive disorders is expected to soar due to an increasing aging population [5]. Cognitive disorders in older people can lead to wide-ranging consequences, including loss of independence, financial misjudgments, economic burden, caregiver burden, increased hospitalizations, overutilization of public resources, and worsening quality of life [6].

There is no cure for neurocognitive disorders or associated cognitive decline [4,7]. Management is, therefore, generally tailored to specific modifiable risk factors, including smoking, hypertension, social isolation, obesity, diabetes, and psychoeducation [8]. Pharmacological approaches focus on distressing behavioral and psychological symptoms alongside cognitive impairment, making psychotropics the mainstay of treatment [7,9]. Cholinergic enhancers like cholinesterase inhibitors slow progression by addressing impairment linked to cholinergic deficits [10]. Antipsychotics, mostly second-generation administered with caution, are the first line for agitation and aggression. At the same time, selective serotonin reuptake inhibitors are recommended for the burdensome neuropsychiatric symptoms of depression and anxiety, and they also serve as alternatives for aggression [11]. Available non-pharmacological treatment options aim to increase or maintain cognitive reserve [12]. Non-pharmacological cognition-focused interventions, either restorative or compensatory, include cognitive stimulation aiding neural plasticity, cognitive training involving guided practice of standard tasks to increase or maintain function as well as minimize impairment, and cognitive rehabilitation offering retraining to improve specific impairments or deficits [12,13]. 

While there are limited treatment options for improving memory in this population, research has shown that special psychotherapeutic modalities like Cognitive Remediation Therapy (CRT), especially when coupled with psychosocial and vocational rehabilitation (PSR and VCR respectively), are beneficial to older adults [14]. Cognitive remediation therapy is a specialized cognitive rehabilitation treatment option with techniques designed to improve neurocognitive abilities with targeted domains, including executive function, attention, planning, cognitive flexibility, and, thus, social functioning [15]. The implementation of this non-pharmacological method results in neuroplasticity, enhanced cognitive reserve, and improved functioning [16]. The maintenance of cognitive ability, either domain-specific or global, is necessary for functional independence. Research has shown the efficacy of various cognitive interventions with reported benefits in older people [17]. In a meta-nalysis of 40 studies that examined the efficacy of CRT on the cognition of patients with schizophrenia, it was found to improve nearly all domains of cognition, and the observed effects were found to be durable [18]. Specifically, CRT has been shown to lead to improvement in memory that benefited social behavior in older adults with schizophrenia compared to younger people with schizophrenia [19], although other studies have shown that younger people benefited more in some domains of cognition [20].

In another meta-analysis of 16 randomized controlled trials (RCT) that examined the efficacy of computer-assisted cognitive remediation (CACR) among schizophrenic patients, significant improvements were noticed in various domains of cognition, including attention, working memory, verbal memory, speed of processing, and social cognition [21]. Ultimately, many more benefits are likely to be derived from a combination of CRT with other forms of therapy. For instance, Lindenmayer et al. (2012) found that when combined with emotional perception remediation, CRT produced improvements in various domains of social functioning, including emotion recognition, emotion discrimination, and neurocognition [22]. A recent systematic review and meta-analysis of 130 studies comprising close to 9000 participants found that in addition to significantly improving cognition and functioning, integration of CRT with psychosocial rehabilitation was integral to the efficacy of the treatments [23]. Despite the documented efficacy of CRT and other non-pharmacological treatment modalities for improving cognition, there is limited study on the availability of CRT and other similar modalities. This study examines the availability of CRT, PSR, and VCR services in US mental health (MH) and substance use (SU) facilities serving older adults and compares the characteristics of the facilities providing these services.

## Materials and methods

Our study used the 2022 National Substance Use and Mental Health Services Survey (N-SUMHSS) data provided by the Substance Abuse and Mental Health Services Administration (SAMHSA). N-SUMHSS data was collected in two parts before 2021: the National Survey of Substance Abuse Treatment Services (N-SSATS) and the National Mental Health Services Survey (N-MHSS). After 2021, the two databases were combined and became N- SUMHSS. The N-SUMHSS survey is an annual survey of public and privately owned mental health and substance use treatment facilities across the US, providing the most comprehensive information about mental health and substance use facilities in the US (SAMHSA website). The 2022 N-SUMHSS data was collected between March 31st, 2023 and December 4th, 2023. Facilities surveyed included psychiatric hospitals, general hospitals, state hospitals, veteran affairs medical centers, certified community behavioral health clinics, partial hospitalization/day treatment facilities, outpatient facilities, residential treatment centers, multi-setting mental health facilities, and community mental health centers. Military MH treatment facilities, unlicensed individual private practitioners/small group practices, and jails or prisons were excluded from the surveys. The questionnaire used in the survey consisted of two screening questions that then decided what part of the questionnaires would be completed by the respondent facilities. The various parts of the questionnaires include (1) the part focused on substance use treatment service, (2) the part focused on mental health treatment services, (3) the part focused on facility characteristics, and (4) the part focused on number of clients in treatment for a given day, month, or 12-month period. There is no information available on the development or validation of the questionnaire used in the survey per the SAMHSA website, and the questionnaires were not designed with any age group in focus as it was designed to collect data from mental health and substance use treatment facilities across the country. Further information on the questionnaire is available at the SAMHSA website (https://www.samhsa.gov/data/system/files/media-puf-file/2022_N-SUMHSS_Questionnaire.pdf).

Data collection was either through a web-based questionnaire, a paper questionnaire sent by mail, or a computer-assisted telephone interview (CATI). Several weeks prior to the survey, facilities received letters from SAMHSA notifying them of data collection and providing them the option of choosing one of the three modalities of data collection. Since data is collected from facilities and not from individuals, with no collection of identifiable personal information, consent from facilities was obtained and not from individuals. Also, information obtained from facilities were not limited to any demographic groups, therefore, there were little to no risks to any vulnerable groups. All data have been de-identified to protect the identities of participants in compliance with the Health Insurance Portability and Accountability Act (HIPAA). Further information about the protection of participants' private information is provided in the SAMHSA N-SUMHSS codebook available online (SAMHSA 2023).

In the statistical analysis, we used the variable "What age groups are accepted for treatment at this facility?" to subset data of participants 65 years and older for the analysis. Variables addressing the availability of CRT, PSR, and VCT services were used to identify facilities providing these services and tabulated against facility-level characteristics such as facility type, ownership, funding source, availability of special programs for veterans, Joint Commission accreditation, availability of supplemental employment services, housing services, recovery housing services, special programs for Alzheimer's disease, and location. Using descriptive statistics, we summarized the characteristics of US mental health and substance use facilities that provide CRT, PSR, and VCR services (available in the next section). In the multivariate logistic regression analysis, we explored facility-level characteristics that predict the availability of CRT, PSR, and VCR services. The availability of CRT, PSR, and VCR services was the outcome variable, while other facility-level characteristics were predictor variables. Some of the statistical assumptions in the multivariate analysis include (1) linearity of the relationship between dependent and independent variables, (2) homoscedasticity, implying constancy of variance of the errors across all levels of independent variables, (3) independence of errors, meaning there is no pattern or correlation between residuals, (4) no multicollinearity, implying that independent variables are not highly correlated with each other, and (5) that the errors (residuals) are normally distributed [24,25]. In this study, cases with missing observations were completely omitted in accordance with recommendations for handling missing data for studies that have large sample sizes and satisfy the missing completely at random (MCAR) assumption [26].

## Results

This study included 1,216 mental health (MH) facilities, with only 78 (6.4%) providing CRT, 381 (31.3%) providing VCT, and 634 (52.1%) providing PSR (Table 1). There were 395 (32%) community mental health centers (CMHC/CCBHC), 413 (34%) outpatient/partial hospitalization facilities, 185 (15%) hospital-based facilities, 108 (9%) Veterans Health Administration (VHA) facilities, 57 (4.7%) residential facilities, and 58 (4.8%) multi-setting/other facilities in the study. Residential facilities had the highest proportion offering CRT (9, 15.8%) and PSR (42, 73.7%), while VHA facilities had the highest proportion offering VCR (80, 74.1%). Nearly nine in 10 (1,042, 86%) of the facilities offered mixed MH and SU services, with about half of these facilities providing PSR (545, 52.3%), a third providing VCR (337, 32.3%), and only a small fraction providing CRT (61, 5.9%). Similarly, among facilities solely providing MH services (174, 14%), approximately half (89, 51.1%) offered PSR, a quarter (44, 25.3%) offered VCR services, and only about one in 10 offered CRT (17, 9.8%).

**Table 1:** Availability of CRT, PSR, and VCR by facility characteristics a: percentages are based on column values; b: percentages are based on row values CRT: Cognitive remediation therapy; PSR: Psychosocial rehabilitation; VCR: Vocational rehabilitation.

Characteristics	Total (n)	%^a^	CRT (n)	%^b^	PSR(n)	%^b^	VCR(n)	%^b^
Facility type								
Community MH Centers (CMHC/CCBHC)	395	32	20.0	5.1	270	68.4	150	38.0
Hospital based	185	15	15.0	8.1	61	33.0	19	10.3
Multi-setting, Others	58	4.8	6.0	10.3	26	44.8	16	27.6
Outpatient/partial hospitalization	413	34	23.0	5.6	173	41.9	93	22.5
Residential	57	4.7	9.0	15.8	42	73.7	23	40.4
VHA	108	8.9	5.0	4.6	62	57.4	80	74.1
Focus (FOCUS)								
MH services	174	14	17.0	9.8	89	51.1	44	25.3
Mixed MH and SU services	1,042	86	61.0	5.9	545	52.3	337	32.3
Ownership								
Private for profit	251	21	29.0	11.6	117	46.6	41	16.3
Private nonprofit/public	965	79	49.0	5.1	517	53.6	340	35.2
Accepts any govt funding								
No (0)	398	33	36.0	9.0	171	43.0	86	21.6
Yes (1)	687	56	32.0	4.7	402	58.5	265	38.6
Don't know (3)	131	11	10.0	7.6	61	46.6	30	22.9
Accepts Medicare								
No (0)	206	17	19.0	9.2	114	55.3	85	41.3
Yes (1)	1,010	83	59.0	5.8	520	51.5	296	29.3
Accepts Medicaid								
No (0)	139	11	18.0	12.9	79	56.8	65	46.8
Yes (1)	1,077	89	60.0	5.6	555	51.5	316	29.3
Special program for Veterans								
No (0)	459	38	16.0	3.5	210	45.8	106	23.1
Yes (1)	757	62	62.0	8.2	424	56.0	275	36.3
Emergency walk-in								
No (0)	608	50	38.0	6.3	263	43.3	143	23.5
Yes (1)	608	50	40.0	6.6	371	61.0	238	39.1
Accreditation Joint Commission								
No (0)	694	57	42.0	6.1	382	55.0	212	30.5
Yes (1)	522	43	36.0	6.9	252	48.3	169	32.4
Offers supply employment services								
No (0)	752	62	54.0	7.2	272	36.2	71	9.4
Yes (1)	464	38	24.0	5.2	362	78.0	310	66.8
Offers housing service								
No (0)	692	57	52.0	7.5	260	37.6	101	14.6
Yes (1)	524	43	26.0	5.0	374	71.4	280	53.4
Recovery housing assist								
No (0)	288	24	21.0	7.3	79	27.4	25	8.7
Yes (1)	928	76	57.0	6.1	555	59.8	356	38.4
Accepts little or no pay for service			0.0					
No (0)	467	38	32.0	6.9	200	42.8	110	23.6
Yes (1)	749	62	46.0	6.1	434	57.9	271	36.2
Offers special program for Alzheimer’s/dementia								
No (0)	904	74	43.0	4.8	490	54.2	280	31.0
Yes (1)	312	26	35.0	11.2	144	46.2	101	32.4
Region (US Regions)								
East North-Central	194	16	15.0	7.7	104	53.6	70	36.1
East South-Central	113	9.3	1.0	0.9	47	41.6	23	20.4
Mid-Atlantic	133	11	9.0	6.8	57	42.9	43	32.3
Mountain	132	11	10.0	7.6	84	63.6	60	45.5
New England	99	8.1	5.0	5.1	32	32.3	34	34.3
Pacific	125	10	9.0	7.2	87	69.6	43	34.4
South Atlantic	217	18	19.0	8.8	108	49.8	51	23.5
West North-Central	71	5.8	2.0	2.8	33	46.5	18	25.4
West South-Central	132	11	8.0	6.1	82	62.1	39	29.5

In the regression analysis (Table 2), compared to community-based facilities, hospital-based facilities (adjusted odds ratio (AOR): 0.40; 95% Cl 0.25-0.65; p<0.001), outpatient/partial hospitalization facilities (AOR: 0.40; 95%Cl 0.27-0.57; p<0.001), VHA facilities (AOR: 0.15; 95%Cl 0.08-0.29; p<0.001), and multi-setting/other types of facilities (AOR:0.43; 95%Cl 0.22-0.84; p=0.014) were significantly associated with lower odds of offering PSR services. In contrast, VHA facilities were significantly associated with higher odds of offering VCR services compared to community-based facilities (AOR:2.17; 95%Cl 1.01-4.78; p=0.050). Facilities that offer special programs for veterans (AOR: 2.25; 95%CI:1.24-4.26; p=0.010) and those that provide special programs for Alzheimer's (AOR 2.49; 95% CI: 1.45-4.26; p=0.001) were more than twice as likely to offer CRT services than those that do not. Publicly owned facilities and those that provide mixed MH and SU services were associated with significantly lower odds of offering PSR (AOR: 0.64; 95%Cl 0.43-0.97; p=0.036) and CRT (AOR: 0.50; 95%Cl: 0.27-0.95; p=0.029) respectively, while those that offer emergency walk-in services were associated with higher odds of offering PSR services (AOR: 1.76; 95%Cl:1.30-2.38; p=0.001). Location-wise, facilities in the East South-Central region of the US were associated with significantly lower odds of providing all three psychotherapeutic modalities examined CRT (AOR: 0.10; 95%Cl:0.01-0.54; p=0.031), PSR (AOR: 0.52; 95%Cl: 0.30-0.91; p=0.022), VCR(AOR: 0.32; 95% Cl:0.15-0.65; p=0.002) while those in the Northeast region were associated with lower odds of providing PSR services (AOR: 0.39; 95% Cl: 0.21-0.70; p=0.002). Facilities in West South-Central and those in the Pacific were associated with lower odds of offering VCR(AOR 0.43; 95%Cl:0.23-0.82; p=0.011) and higher odds of offering PSR (AOR:2.51; 95%Cl:1.44-4.42; p=0.001) respectively. 

**Table 2:** Adjusted associations between facility-level characteristics and availability of CRT, PSR, and VCR AOR: Adjusted odds ratio; CI: Confidence interval; CRT: Cognitive remediation therapy; PSR: Psychosocial rehabilitation; VCT: Vocational rehabilitation; *p-*value: Level of significance; ref: Reference

Characteristics	CRT			PSR			VCR		
	AOR	95%CI	*p*-value	AOR	95%CI	*p*-value	AOR	95%CI	*p*-value
Facility type (ref									
Hospital based	0.72	0.30,1.69	0.459	0.40	0.25,0.65	< .001>	0.92	0.45,1.81	0.811
Multi-setting, Others	1.33	0.43,3.65	0.594	0.43	0.22,0.84	0.014	1.26	0.55,2.83	0.580
Outpatient/partial hospitalization	0.65	0.31,1.27	0.195	0.40	0.27,0.57	< .001>	0.76	0.49,1.19	0.226
Residential	2.08	0.71,5.80	0.170	1.32	0.64,2.83	0.455	1.99	0.86,4.53	0.102
VHA	0.29	0.73,0.96	0.054	0.15	0.08,0.29	< .001>	2.17	1.01,4.78	0.050
Ownership (ref: Private for profit)									
Private nonprofit/public	0.61	0.31,1.20	0.149	0.64	0.43,0.97	0.036	1.37	0.78,2.44	0.279
Focus (ref: MH services)									
Mixed MH and SU services	0.50	0.27,0.95	0.029	0.73	0.49,1.08	0.114	0.82	0.50,1.34	0.422
Accepts any govt funding (ref: No)									
Yes (1)	0.82	0.42,1.60	0.555	1.14	0.80,1.64	0.468	0.88	0.56,1.37	0.564
Don't know (3)	1.12	0.46,2.58	0.793	1.33	0.81,2.17	0.259	0.63	0.32,1.21	0.171
Accepts Medicare (ref: No)									
Yes (1)	0.91	0.41,2.12	0.816	0.94	0.60,1.49	0.796	0.74	0.40,1.39	0.346
Accepts Medicaid (ref: No)									
Yes (1)	0.43	0.18,1.02	0.052	0.67	0.39,1.15	0.144	0.82	0.39,1.77	0.602
Special program for Veterans (ref: No)									
Yes (1)	2.25	1.24,4.26	0.010	1.14	0.84,1.54	0.395	1.00	0.69,1.45	0.986
Offers special program for Alzheimer’s/dementia (ref: No)									
Yes (1)	2.49	1.45,4.26	0.001	0.95	0.68,1.33	0.771	1.15	0.74,1.78	0.538
Offers supply employment services (ref: No)									
Yes (1)	1.13	0.59,2.13	0.705	4.60	3.31,6.45	< .001>	15,01	10.32,22.18	< .001>
Offers recovery housing (ref: No)									
Yes (1)	0.99	0.54,1.84	0.966	1.99	1.40,2.84	< .001>	2.23	1.35,3.79	0.002
Offers housing service (ref: No)									
Yes (1)	0.57	0.30,1.08	0.086	1.96	1.41,2.72	< .001>	2.09	1.43,3.06	< .001>
US Regions (ref: Midwest)									
East South-Central	0.10	0.01,0.54	0.031	0.52	0.30,0.91	0.022	0.32	0.15,0.65	0.002
Mid-Atlantic	0.73	0.28,1.82	0.501	1.09	0.64,1.86	0.741	1.57	0.81,3.03	0.179
Mountain	0.98	0.38,2.89	0.957	1.45	0.85,2.49	0.175	1.32	0.71,2.47	0.381
New England	0.58	0.18,1.65	0.334	0.39	0.21,0.70	0.002	0.92	0.46,1.83	0.811
Pacific	0.83	0.31,2.16	0.709	2.51	1.44,4.42	0.001	0.64	0.33,1.23	0.181
South Atlantic	1.00	0.47,2.19	0.992	1.35	0.85,2.15	0.204	0.72	0.40,1.30	0.272
West North-Central	0.33	0.05,1.26	0.155	0.72	0.37,1.40	0.336	0.53	0.23,1.19	0.126
West South-Central	0.68	0.25,1.70	0.417	1.37	0.79,2.38	0.264	0.43	0.23,0.82	0.011
Emergency walk-in services (ref: No)									
Yes (1)	1.28	0.74,2.22	0.382	1.76	1.30,2.38	0.001	0.98	0.68,1.41	0.904

## Discussion

Our analysis found that only 6% of the facilities included in this study offered CRT. While most US MH and SU facilities (more than one-third of facilities in this study were community mental health centers (CMHC/CCBHC) and outpatient/partial hospitalization facilities) are community-based [27], our study found that these types of facilities offered CRT the least compared to other types of facilities. The older population in the United States is fast-growing, making up about 17% of the general population in 2022, and is projected to increase by 53.6% between 2022 and 2060 [28]. Meanwhile, 1-2% of older people are estimated to be affected by major cognitive disorders by age 65 and up to 30% by age 85. Sustained advancement in healthcare is expected to lead to better life expectancy, meaning more older people will live up to 85 years and more. Therefore, it should be expected that the proportion of people suffering from neurocognitive disorders, and therefore needing some forms of therapies to improve cognition, will increase exponentially. Further, research has shown that CRT is not only indicated in elderly with mild to moderate neurocognitive disorders but has the potential to at least reverse cognitive decline in the population [29]. This is partly because the potential for neuroplasticity has been found to persist even in advanced age [29]. Despite the apparent utility of cognitive enhancing modalities, these services are not widely available. Community-based centers are best positioned to serve the elderly population and so should be equipped with these kinds of services. 

Our finding of the relative unavailability of CRT and related services is consistent with findings from various studies that continue to report the relative unavailability of CRT services despite the proven efficacy of the approach [30,31]. Some of the suggested reasons for this scarcity include a lack of (1) recognition of the importance of cognitive health, (2) staff with adequate training in CRT services, (3) necessary infrastructure to support CRT implementation, (4) clear guidelines on implementation strategies, and (5) sufficient enthusiasm towards CRT benefits [32]. Wykes and colleagues documented the efficacy of CRT more than 10 years ago, envisioning that there would be widespread implementation of the modalities across mental health facilities [33]. Today, that optimism seems evasive given their realization that substantial technical and logistic barriers to the implementation of cognitive remediation approaches persist, prompting their suggestion for more research and calling for increased interest among MH providers in the understanding of how cognitive remediation therapies benefit patients [30]. More specifically, many mental health professionals are unaware of the need to target cognitive function in patients with disorders that are associated with cognitive decline. Also, mental health authorities are yet to provide definitive guidelines to guide providers in implementing cognitive remediation modalities, and as a result, there are insufficient trained professionals to implement CRT [31,34]. Given that various research on CRT in the geriatric population continue to report substantial variability in CRT outcomes related to age and other factors, there is a need for more research into the utility of CRT among this population as well as a need to develop clear guidelines on the application of CRT in the geriatric population. This will further substantiate claims of effectiveness in improving cognitive abilities in older people and increase clinicians’ enthusiasm in recommending and providing CRT services.

Another finding from this study was that publicly owned facilities and those that provide mixed MH and SU services were associated with significantly lower odds of offering PSR and CRT services, respectively. One factor that could explain this finding may be related to funding. For instance, compared to for-profit, privately-owned facilities, publicly owned facilities may have less access to funds as they are likely to charge payers less [35]. With more funding, privately owned facilities may be well positioned to be able to afford the resources required to provide these services, compared to publicly owned facilities. Private for-profit facilities make up a very small proportion of US mental health and substance use facilities, according to data from SAMHSA; therefore, they likely serve a minority of the US population [36]. This implies that reduced availability of CRT and PSR services in public/non-for-profit facilities compared to private for-profit facilities may translate to further reduction in access to these services among most older Americans. Although many publicly owned facilities may offer other psychotherapeutic options such as PSR/VCT or neuromodulation, studies have shown that a combination of CRT with PSR or other modalities leads to stronger psychosocial functioning than either standalone CRT or PSR [37]. Furthermore, many modalities that use neuromodulation are not widely available, especially in publicly funded facilities [38].

Another significant finding from our study was those facilities in the East South-Central region of the US, comprising states of Alabama, Tennessee, Mississippi, and Kentucky, were less likely to provide all three psychotherapeutic modalities investigated (CRT, PSR, and VCT) compared to facilities in the Midwest region of the US (Indiana, Illinois, Michigan, Ohio, Wisconsin, Iowa, Kansas, Minnesota, Missouri, Nebraska, North Dakota, and South Dakota). The inherently limited research in nonpharmacologic therapies for cognitive decline and their availabilities makes it difficult to explain the observed state-to-state variabilities in the availability of these services.

## Conclusions

This study examined a critical subject of the availability of non-pharmacological treatment alternatives for older adults with cognitive deficits, specifically cognitive remediation therapy. At a time when life expectancy has significantly improved, and older people are expected to make up a significant portion of the population, cognitive decline could be a major burden to healthcare, other members of society, and the economy. The scarcity of resources to address cognitive decline among older people is a preventable problem that should be addressed before it spirals out of control. Health authorities and policy-makers should therefore be proactive and enact legislation and provide programming that would ensure widespread availability of non-pharmacological options for addressing cognitive decline in the older population. This is even more important given that older people are likely to be on multiple medications for multiple health conditions, making nonpharmacological alternatives very important.
